# Soil gas probes for monitoring trace gas messengers of microbial activity

**DOI:** 10.1038/s41598-021-86930-8

**Published:** 2021-04-15

**Authors:** Joseph R. Roscioli, Laura K. Meredith, Joanne H. Shorter, Juliana Gil-Loaiza, Till H. M. Volkmann

**Affiliations:** 1 Aerodyne Research, Inc., Billerica, MA 01821 USA; 2 School of Natural Resources and the Environment, University of Arizona, Tucson, AZ 85721 USA; 3 University of Arizona, Biosphere 2, Oracle, AZ 85623 USA; 4 Applied Intelligence, Accenture, Kronberg Im Taunus, 61476 Hesse, Germany

**Keywords:** Biogeochemistry, Environmental sciences, Environmental chemistry, Environmental monitoring, Biogeochemistry, Element cycles, Soil microbiology, Microbial ecology

## Abstract

Soil microbes vigorously produce and consume gases that reflect active soil biogeochemical processes. Soil gas measurements are therefore a powerful tool to monitor microbial activity. Yet, the majority of soil gases lack non-disruptive subsurface measurement methods at spatiotemporal scales relevant to microbial processes and soil structure. To address this need, we developed a soil gas sampling system that uses novel diffusive soil probes and sample transfer approaches for high-resolution sampling from discrete subsurface regions. Probe sampling requires transferring soil gas samples to above-ground gas analyzers where concentrations and isotopologues are measured. Obtaining representative soil gas samples has historically required balancing disruption to soil gas composition with measurement frequency and analyzer volume demand. These considerations have limited attempts to quantify trace gas spatial concentration gradients and heterogeneity at scales relevant to the soil microbiome. Here, we describe our new flexible diffusive probe sampling system integrated with a modified, reduced volume trace gas analyzer and demonstrate its application for subsurface monitoring of biogeochemical cycling of nitrous oxide (N_2_O) and its site-specific isotopologues, methane, carbon dioxide, and nitric oxide in controlled soil columns. The sampling system observed reproducible responses of soil gas concentrations to manipulations of soil nutrients and redox state, providing a new window into the microbial response to these key environmental forcings. Using site-specific N_2_O isotopologues as indicators of microbial processes, we constrain the dynamics of in situ microbial activity. Unlocking trace gas messengers of microbial activity will complement -omics approaches, challenge subsurface models, and improve understanding of soil heterogeneity to disentangle interactive processes in the subsurface biome.

## Introduction

Soil microbiomes influence ecosystem health and interact with changing environments by governing biogeochemical cycles. Microbial processes involve chemically diverse gases, e.g., carbon dioxide (CO_2_), methane (CH_4_), nitrous oxide (N_2_O), and volatile organic compounds (VOCs), that are fundamental to the role of soils in carbon sequestration, nutrient use efficiency, and greenhouse gas emissions^1,2^. The gas cycling functions of microbes are increasingly predicted from the phylogenetic diversity and functional potential of soil microbiomes^1^, however, measuring the associated rates of microbial gas cycling processes in situ remains a technical challenge. New tools that quantify subsurface biogeochemical cycling are needed to constrain critical soil functions at spatiotemporal scales relevant to soil microbial communities.

Spatially resolved subsurface gas measurements help disentangle the contributions of subsurface microbial processes to the net gas exchange at the soil surface^3^. Multiple, potentially competing, biogeochemical processes occur in soil and depend upon microbial composition, resources, and environmental conditions that are heterogenous on both spatial^4,5^ and temporal scales^6–8^. The resulting inhomogeneity of the rates of microbial processes in space (hot spots) or time (hot moments) contributes significantly to net soil function^9,10^, for example during rapid fluctuations in soil conditions (e.g., rapid rewetting^11^) or in proximity to plant root zones^12^. While small-scale soil processes and structure (µm to mm) have important roles in biogeochemical cycling, they are difficult to address with above-ground soil flux measurements at larger scales, e.g., soil chamber (cm^2^) or tower (m^2^ to km^2^). In addition, these same soil processes can vary on temporal scales that are challenging to resolve with genomic and other multi-omic approaches that produce rich yet discontinuous ‘snapshots’ into soil microbial communities and function. Soil gas measurements at relevant spatiotemporal scales are critical to bridge the gap between the resolution provided by -omics and real-world heterogeneity in soil.

There is a growing interest in leveraging recent advances in trace gas sensing technology to directly quantify subsurface interstitial gases in real time with minimal disruption. Previous extractive soil gas sampling approaches inherently drove advective mass flow of gas in soil, which disproportionately favors gas in large pores, and may draw sample from afar along preferential flow paths^13–15^. Later approaches used sampling devices that pre-equilibrated sampling volumes with soil gas by diffusion into open tubes^16,17^ or traps^18,19^ with sufficient volume to withdraw sample gas while reducing contamination by advective mass flow. However, these devices have the disadvantage of a larger spatial footprint, with few exceptions^20^, and potential for bulk water contamination. This approach was improved by allowing soil gas to diffuse into an internal sampling volume across barrier membranes composed of silicone^21^, polypropylene^22–24^, polyethylene^25^, or expanded PTFE (ePTFE)^26–29^. Although diffusive membranes have been integrated into online gas sampling systems to study soil, water, and plant gas cycling (e.g. ^24,30–33^), identifying materials that satisfy the multiple requirements for deployment in the soil system has limited the impact of the approach. Probe membranes must efficiently promote gas diffusion while having the following attributes: resistant to bulk advective flow^22^; hydrophobic to prevent liquid water passage; chemically and biologically inert (i.e., resist biofouling^34^); and structurally robust (i.e., withstand freezing^22^ or crushing^27^). Recent developments in porous materials show promise for developing diffusive membranes that satisfy these requirements and can be integrated into small-scale probes for minimally disruptive online sampling of soil gases.

Soil gas composition is diverse, but few chemical species have been measured with online soil gas sampling, despite significant advances in trace gas analysis. Self-contained and field-robust in situ (i.e., buried) gas analyzers exist only for a limited selection of gases (e.g., CO_2_^35–37^ and O_2_^38,39^), making sample transfer from diffusive soil probes to aboveground trace gas analyzers the most viable online approach for most gases involved in microbial processes. The diffusive sampling approach has been used to measure gases from soil and other samples in real time by gas chromatography (e.g., N_2_O, nitric oxide (NO), CO_2_, and Radon-222^22^), CO_2_ infrared gas analyzers^27^ and sensors^40^, and, more recently, laser spectrometers for stable isotopologues of water vapor and CO_2_ (e.g. ^30,41^). Expanding the suite of trace gases that can be measured in the subsurface will increase the fraction of the volatile metabolome (volatilome) of microbial communities and their biological interactions accessible for study. Many biologically interactive small molecules and their isotopologues (e.g., N_2_O, CH_4_) are now routinely monitored in the gas phase by direct absorption spectroscopy and online mass spectrometry with excellent precision and time resolution. These recently developed analytical tools can be modified and coupled with optimized subsurface diffusive probe designs to yield new and deeper insights into a growing range of biogeochemical processes.

**Figure 1:**
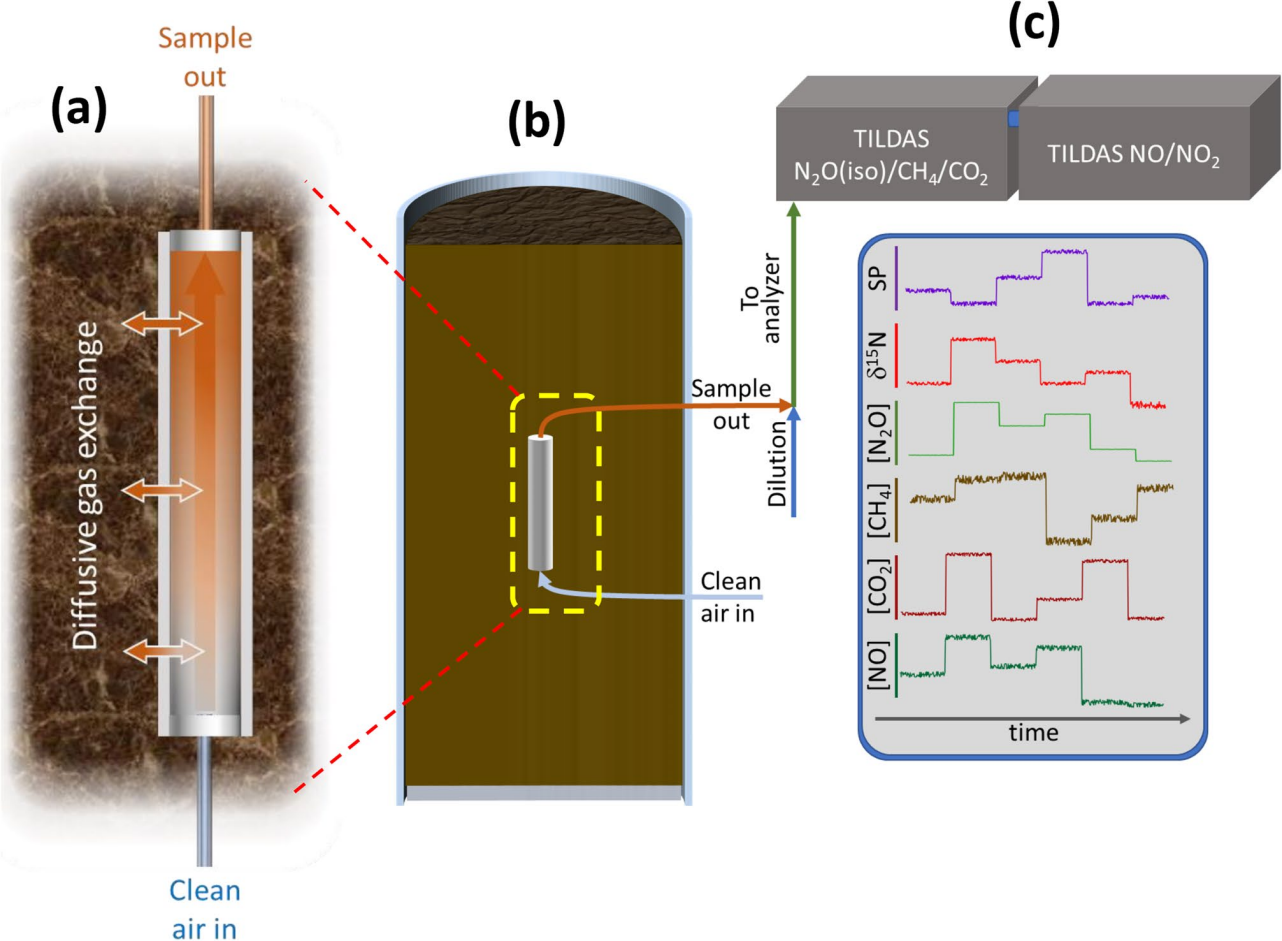
(**a**) Diffusive exchange across sPTFE membrane. (**b**) Probe mounted in the soil column. Sample air exiting the probe is diluted and transferred to analyzers (**c**) that measure time series of concentrations and isotopic ratios from multiple probes.

Here we demonstrate the potential of a new soil gas measurement system that illuminates subsurface nutrient cycling by coupling a minimally disruptive soil gas sampling approach with online analyzers, to yield high temporal resolution on spatial scales that start to approach high-resolution offline gas probes^20^. While previous systems have successfully measured soil gas composition at high spatial and temporal resolution, achieving both for many trace gases has been elusive. Advances in probe design, gas analyzers, and flow schemes described here (Materials and Methods) unlocks a wide range of trace gas instrumentation for real time, non-invasive tracking of biogeochemical processes^42^. We constructed novel diffusive probes from porous sintered PTFE (sPTFE) (Fig. 1a)—a material with superior hydrophobicity, inertness, cleanability, microfiltration, and pore distribution uniformity to previous probe membranes^43^. We coupled these probes to multiple Tunable Infrared Laser Direct Absorption Spectroscopy (TILDAS) analyzers (Fig. 1c) to target trace gas messengers of nitrogen and carbon cycling (N_2_O, NO, nitrogen dioxide (NO_2_), CH_4_, CO_2_), and the site-specific stable isotopologues of N_2_O, whose signatures reflect N_2_O production pathways^44,45^. In this study, we demonstrate the approach using controlled soil columns (Fig. 1b) while manipulating soil nutrient and redox conditions. These techniques represent a general approach to monitor trace gases as messengers of soil microbial processes to help interpret and model the effects of critical soil microbiomes.

## Results and discussion

The performance of the diffusive sampling system based upon hydrophobic sPTFE subsurface gas probes (Fig. 1) was demonstrated in two mesocosm experiments: 1) addition of fertilizer to three columns of identical topsoil under ambient redox conditions, resolving different temporal responses among N_2_O, NO, CH_4_ and CO_2_ trace gas messengers; and 2) addition of fertilizer to two topsoil columns under different redox environments, clocking the onset of anaerobic methanogenesis and aerobic nitrification, and identifying methane “hot moments”. These studies underscore the potential of in situ gas measurements to provide new insights into nutrient cycling dynamics on fast timescales. They also show that the diffusive probe approach recovers trace gas concentration dynamics that are reproducible and representative of expected subsurface microbial activity.

### Experiment 1: dynamics of N consumption

**Figure 2:**
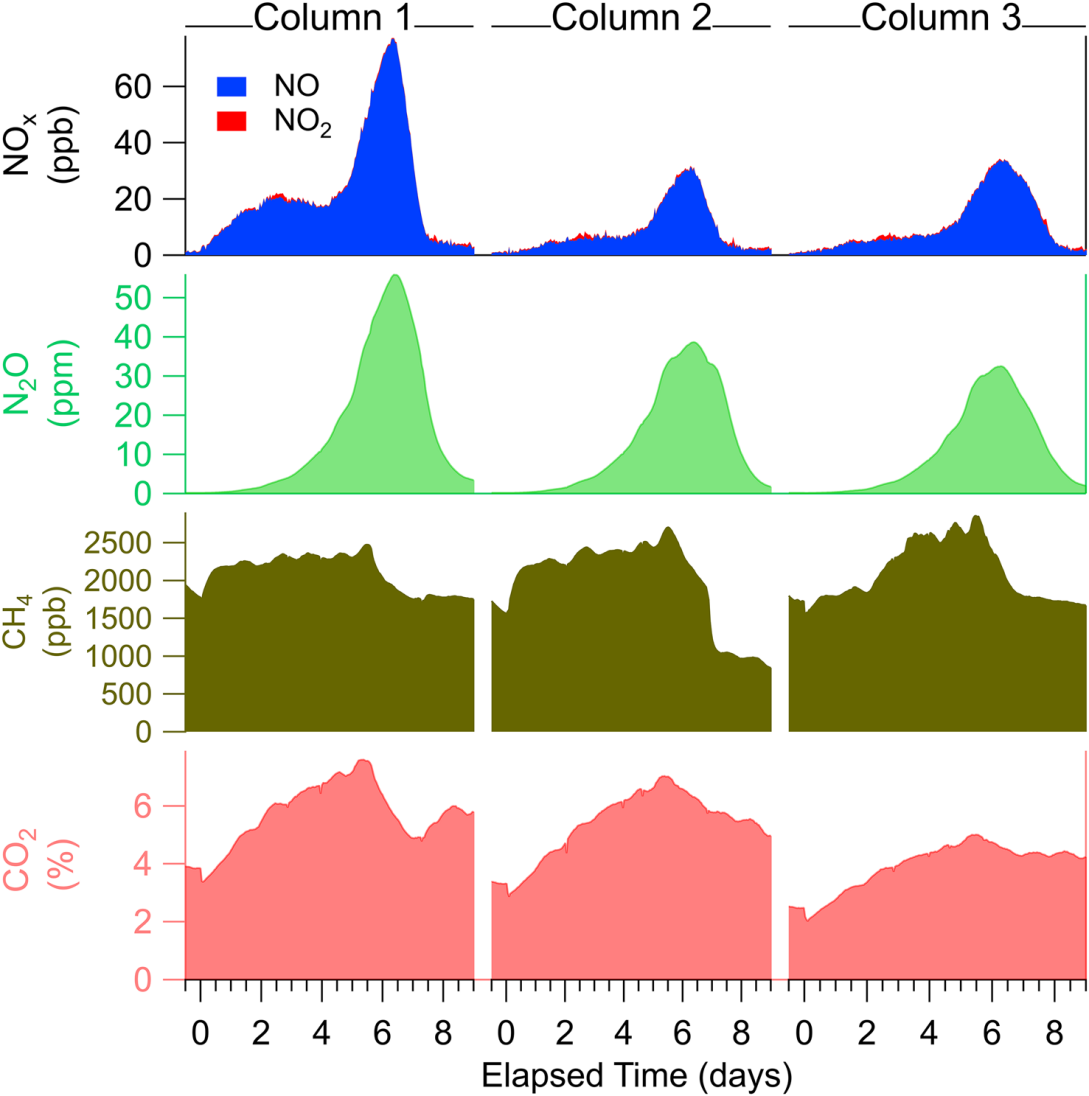
Temporally resolved subsurface gas concentrations show dynamic, reproducible responses to nitrogen fertilizer addition (at time zero) in three replicate soil columns of Experiment 1. Total NO_x_ (top panel) is the sum of NO (blue) and NO_2_ (red).

Reproducible, temporally-resolved nutrient-cycling dynamics were observed in soil columns in response to controlled fertilization treatments that we expected would increase production of N-containing trace gases. N_2_O concentrations exhibited a delayed peak approximately 6 days after N addition (Fig. 2)^46,47^, rising from ambient levels (0.325 parts per million, ppm) to a maximum of 30–55 ppm. A similar response in N_2_O was observed in all columns: an exponential rise in concentration (doubling time of 0.82(0.03) days; average across replicates (sd)), a peak at 6.34(0.06) days, and a rapid fall after the peak (halving time of 0.51(0.06) days). CO_2_ levels also increased concomitantly with the N_2_O pulse and decreased with the falling edge (after day 6), although with qualitatively different temporal signatures (slow rise and fall over the course of the N_2_O pulse). High CO_2_ concentrations (4–7%, v/v) indicated active microbial respiration. In contrast, CH_4_ concentrations were near ambient levels (~ 1.9 ppm CH_4_) during the N_2_O pulse, but decreased after day 6 to sub-ambient levels, indicating methanotrophic consumption. Signatures of microbial respiration and methanotrophy indicate an abundance of aerobic activity in Experiment 1, but do not rule out the possibility of anaerobic sites in the soil.

Combining multiple, fast trace gas instruments provided a comprehensive view of subsurface nutrient cycling. Nitric oxide (NO) is a critical intermediate in nitrification and denitrification, and its emission from soil is a major contributor to budgets of nitrogen oxides (NO_x_) in certain regions^48^. In response to N addition, fast NO measurements showed two features (Fig. 2): an initial rise within 12 h of N addition that preceded N_2_O, and a second rise concomitant with N_2_O. NO_2_ concentrations were over twenty times smaller than NO, and exhibited no correlation with N addition, indicating that NO_2_ was not a significant source of subsurface NO_x_ in this system. NO-producing pathways are evidently activated by N addition before N_2_O-producing pathways, suggesting that these fast, in situ probe measurements can temporally resolve at least two active nitrogen transformation pathways^49^.

**Figure 3:**
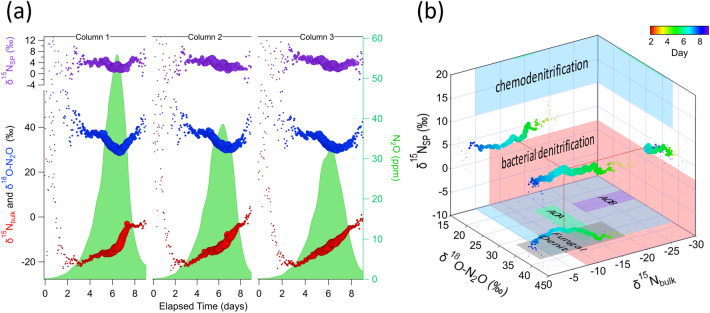
N_2_O pulse responses to N addition were accompanied by reproducible trends in N_2_O isotopes. (**a**) N_2_O concentration (green shading) pulses and shifts in bulk δ^15^N_bulk_ (red), δ^18^O-N_2_O (blue), and δ^15^N_SP_ (purple) in replicate soil columns (Experiment 1). Marker size reflects N_2_O concentration, and precision of N_2_O isotope signatures increased with N_2_O concentration. (**b**) Projection of bulk δ^15^N_bulk_ (x-axis), δ^18^O-N_2_O (y-axis), and site preference (z-axis) probe measurements onto regions of known microbial N_2_O pathways. On the x–y axis AOA (green rectangle) and AOB (purple rectangle) refer to NH_3_ nitrification from ammonia oxidizing archaea ^56,72^ and ammonia oxidizing bacteria ^50,56,73^, respectively. Grey rectangle indicates fungal denitrification ^74^. Uncertainties in isotopic values due to matrix effects and calibration drift are 5.0‰, 1.6‰, and 2.5‰ for δ^15^N_bulk_, δ^15^N_SP,_ and δ^18^O-N_2_O respectively.

Isotopic signatures of N_2_O—^14^N^15^N^16^O (δ^15^N_α_), ^15^N^14^N^16^O (δ^15^N_β_) and ^14^N^14^N^18^O (δ^18^O-N_2_O)—serve as indicators of microbial nutrient cycling pathways and dynamics. Previous work established that ^15^N_bulk_ (^15^N_bulk_ = (δ^15^N_α_ + δ^15^N_β_)/2), and δ^18^O-N_2_O reflect both N_2_O production pathway and substrate ^15^N (or ^18^O) content. Site preference (δ^15^N_SP_)—the preference for the ^15^N to be on the central (“α” position) versus end (“β” position) of the N_2_O molecule (δ^15^N_SP_ = (δ^15^N_α_–δ^15^N_β_))—only reflects the production pathway^44^. In this study ^15^N_bulk_ values shifted with reproducible patterns (initial drop followed by rise across N_2_O pulse) during the N_2_O pulse observed in Experiment 1 (Fig. 2). Changes in the site preference (δ^15^N_SP_) were nearly identical across the three columns (Fig. 3), dropping over the course of the N_2_O pulse from 4.7(0.6)‰ on days 3–5 to 3.2 (0.6)‰ on days 7–8. δ^18^O-N_2_O was stable at 37.7(1.6)‰ until day 5, when it decreased, eventually reaching < 33‰ by day 7. These isotopic signatures (δ^15^N_bulk_, δ^15^N_SP_, δ^18^O-N_2_O) along with concentrations of N-cycling intermediates provide a palette of biogeochemical messengers that unravel the time-dependent contributions of different processes that comprise the soil microbiome response to N addition.

The dynamics of δ^15^N_bulk_ isotopic signatures reflect the consumption rate and progressive exhaustion of the subsurface nutrient pool. After nitrogen addition, we observed a rapid reduction in δ^15^N_bulk_ in all columns to - 20.0 (1.1)‰ on days 2–3 (Fig. 3), followed by an increase for the remainder of the N_2_O pulse. Generally, microbial processes generate NO_2_^-^, NO_3_^-^, N_2_, NO, and N_2_O depleted in ^15^N relative to ^14^N as compared to the substrate, which results in ^15^N *enrichment* of the remaining soil N substrate (here fertilizer)^50^. We observed increasing δ^15^N_bulk_ during the N_2_O pulse in all columns (3.4(0.2)‰/day from day 4–7.5), indicating ^15^N enrichment of the substrate by N consumption routes. The reduction in slope near day 8 suggests a slowing of processes that enriched the substrate, consistent with the N pool being nearly consumed. The δ^15^N_bulk_ shift therefore effectively clocks substrate consumption from N_2_O production as well as other routes. Future work will incorporate the observed δ^15^N_bulk_ into a kinetic model framework to quantify the fractionation associated with other consumption routes that are reflected in the observed δ^15^N_bulk_ slope.

Site-specific stable isotopologues of N_2_O reflect microbial N_2_O production and consumption pathways. Previous measurements of N_2_O isotopic signatures (δ^15^N_bulk_, δ^15^N_SP_ and δ^18^O-N_2_O) of key N_2_O pathways and biological groups (i.e., bacteria, fungi, archaea) have outlined signature regions for microbial N_2_O processes (Fig. 3b)^44^. In this experiment, δ^15^N_SP_, δ^15^N_bulk_, and δ^18^O-N_2_O values indicated that the produced N_2_O was derived from both bacterial denitrification^50–53^ and (abiotic) chemodenitrification^51,54,55^. Notably, nitrification signatures exhibit δ^15^N_SP_ values > 20‰, well outside of the range of values observed here^50,52,56^. However, N_2_O production may reflect contributions from multiple pathways, which may not be separable using isotopic signatures alone. While the observed isotopic data provide evidence for bacterial denitrification and chemodenitrification, other pathways including bacterial nitrification, N_2_O reduction, and archaeal and fungal N-cycling may also contribute.

The combined measurements of isotopic signatures and other N cycling intermediates provide deeper insight into the multiple processes that contribute to N pulse dynamics and N cycling. In this case, changes in NO vs. N_2_O concentrations, along with the changes in δ^18^O-N_2_O and δ^15^N_SP_ near day 5, are consistent with a picture of shifting contributions from chemodenitrification and bacterial denitrification during the experiment. First, the observed change in δ^18^O-N_2_O on day 5 indicated a shift in N_2_O production pathway resulting in different ^18^O/^16^O fractionation, and/or access to a different O substrate (e.g., NO_2_^−^ vs NO_3_^−^)^44,57^. Second, the reduction in δ^15^N_SP_ value, correlated with δ^18^O-N_2_O as shown in Supplemental Figure S5^50^, suggested that the shift in pathway was toward bacterial denitrification. Finally, chemodenitrification is known to produce primarily NO^58–61^, while bacterial denitrification produces both NO and N_2_O as intermediates. The observed early NO enhancement (day 0–5, Fig. 2) that was uncorrelated with N_2_O therefore indicated the presence of chemodenitrification. After day 5 an increase in bacterial denitrification yielded correlated NO and N_2_O signals (Supplemental Figure S6). The picture emerging from these trace gas messengers is that chemodenitrification was active soon after N addition, while bacterial denitrification did not develop until several days later. Further analysis of soil chemical (e.g., nitrate and nitrite content) and biological (e.g., via genetic profiling) properties would add additional context to these observed fast trace gas and isotopic trends and help untangle the contributions of myriad N-cycling processes. These results demonstrate the potential for this approach to provide temporal resolution that could complement these chemical and -omics approaches to provide deeper insights into both community function and dynamics.

### Experiment 2: redox manipulation

**Figure 4:**
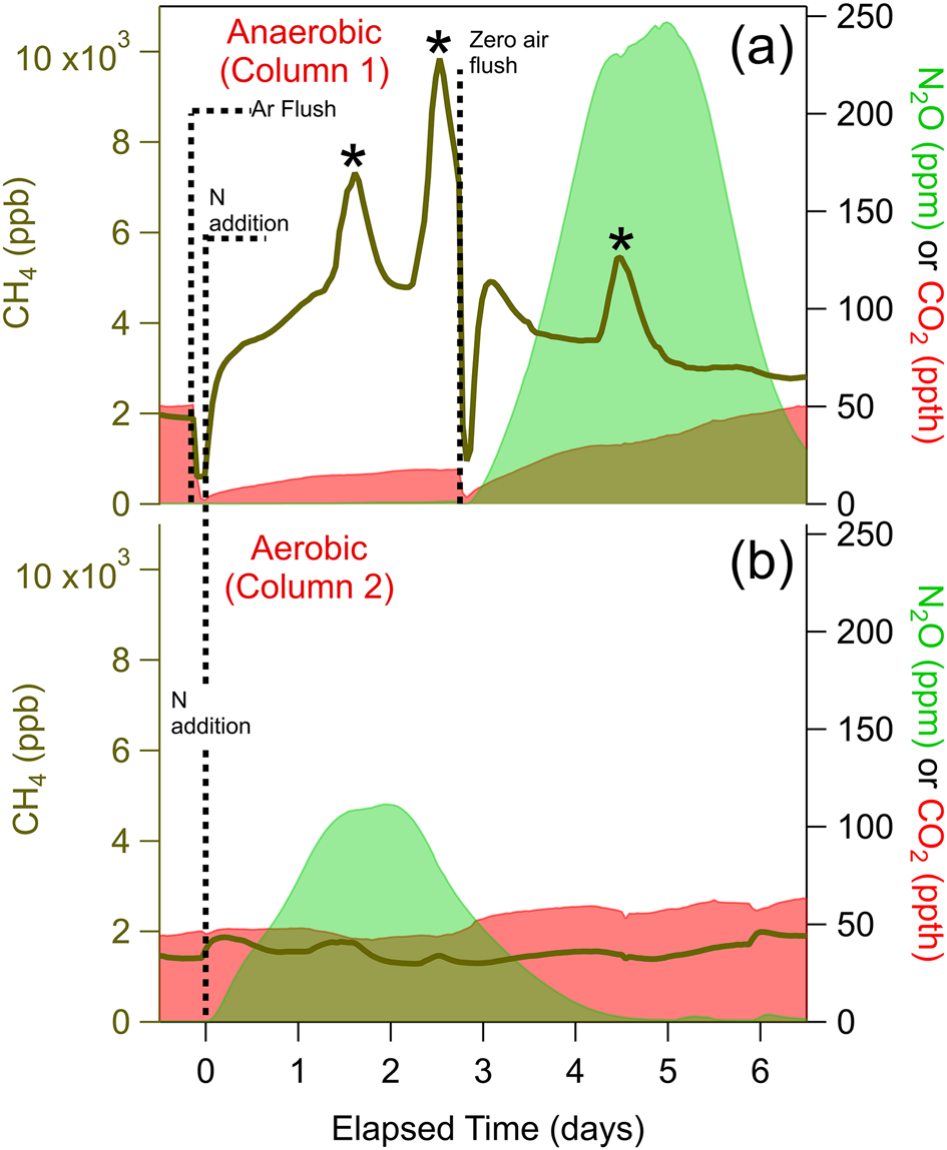
Response of soil N_2_O (shaded green, ppm), CH_4_ (brown line, ppb), and CO_2_ (shaded red, parts per thousand) concentrations to inorganic nitrogen addition (day 0) under (**a**) anaerobic and (**b**) aerobic conditions. The anaerobic column was maintained under Argon until an ultra zero air flush on day 3 (Supplemental Table S1). “Hot moments” of CH_4_ are marked with *.

In Experiment 2, we used diffusive probes to provide an undisturbed view of microbial response to environmental redox shifts. We tracked soil N_2_O, CO_2_, and CH_4_ in response to reduced N addition in columns under different redox conditions: anaerobic (flushed with Argon) to aerobic (Fig. 4a) *vs.* consistently aerobic (Fig. 4b). As in Experiment 1, the aerobic soil column conditions supported N_2_O production after fertilizer addition. In contrast, under forced anaerobic conditions, oxidation of reduced fertilizer NH_4_^+^ to NO_3_^−^ and NO_2_^−^ by nitrification was inhibited, halting N_2_O production by nitrification and subsequent denitrification. Thus, anaerobic N_2_O production was delayed and only began after oxygen was reintroduced 3 days later. Compared to the aerobic column CO_2_ (5%; 50 parts per thousand, ppth), anaerobic column CO_2_ concentrations were lower (< 1%), consistent with persistent anaerobic conditions.

The high temporal resolution of probe measurements revealed differences in subsurface trace gas dynamics. CH_4_ concentrations increased above ambient levels (1.9 ppm) within 5 h of forcing anaerobic conditions, indicating rapid onset of obligately anaerobic methanogenesis. The observed subsurface CH_4_ remained elevated for 8 days following the return to aerobic conditions (on day 3), indicating the persistence of anaerobic centers in the soil or a slow deactivation of methanogenic pathways. In contrast to the slow responses of CH_4_ and CO_2_, N_2_O responded immediately to the sudden aerobic conditions, producing an intense pulse that peaked 2.2 days after introduction of aerobic conditions, with full width at half maximum of 2.0 days.

Fast subsurface gas measurements can complement surface flux measurements by providing an in situ view into “hot spots” and “hot moments”, which might be particularly difficult to capture with offline, manual sampling approaches. Under anaerobic conditions, methane concentrations exhibited large transient increases (marked by * in Fig. 4a) between 6 and 12 h in duration that were not observed in the other monitored soil gases. Transient peaks may be indicators of methanogenic “hot moments” of CH_4_ surface emissions, as previously observed by aboveground flux approaches^62–64^. Under a continuous aerobic environment (Fig. 4b), no methanogenic “hot moments” were observed in CH_4_ or other gases. A “hot moment” was also observed 2.5 days after reintroduction of aerobic conditions (Fig. 4a), indicating these events are possible during recovery from anaerobic conditions.

## Conclusions

We demonstrated technical advances that track microbial activity, nutrient cycling, and dynamic subsurface responses to environmental conditions. To enable real-time, spatiotemporally resolved measurements of diverse soil gases, we coupled high-precision online trace gas analyzers and diffusive soil gas probes in ways that begin to level the playing field compared to low-cost or low-volume measurement methods. This innovation required new developments in soil gas sampling, transfer, and detection. Specifically, we modified a novel TILDAS and developed a controlled sample transfer scheme (online dilution) that reduce the volume required to sample soil gas via diffusion-based probes. These lower volumes minimize overall sampling disruption (less gas exchange), allowing for higher probe sampling rates, and smaller probes that start to approach the spatial scale of low-volume offline soil gas sampling systems. We coupled this system to novel, low-profile, rugged diffusive sPTFE probes that exhibit chemical and biological inertness and long-term hydrophobicity. Finally, we demonstrate approaches (spectral, dilution, and correction factors) for overcoming analytical challenges associated with adapting atmospheric trace gas analyzers to the soil system. Together, these changes strike a balance between temporal and spatial resolution that has been out of reach for many soil gases and isotopologues. Looking forward, these advances offer exciting possibilities to complement and bridge spatiotemporal gaps of current methods for a variety of trace gases and subsurface systems.

### Spanning spatiotemporal disconnects

Continued improvement of trace gas analyzers to detect new species with increased sensitivity and precision will expand capabilities in microbial tracking using diffusive probes. Subsurface processes are heterogenous on many scales, from microns to meters. Higher sensitivity will help reduce required sample size and probe dimensions, leading to measurements at smaller scales that, for example, resolve the influence of soil gases on rhizosphere processes and interactions^12^. In addition, continued coordinated improvements in reduced sample size and sample transfer design could enable sampling approaches similar to those with low-volume analyzers and sensors (e.g., closed-loop recirculation^40,65^ and equilibrated sample volumes passed intact to an analyzer), that further help reduce the impact of sampling on subsurface gas composition and chemistry. Detection of new species (e.g., nitrification intermediate hydroxylamine) and isotopologues (e.g., ^15^NO), will provide further constraints on microbial pathways and dynamics. Coupled to arrays of diffusive soil gas probes, these capabilities will enable real-time three-dimensional mapping of subsurface chemical profiles, to yield new insights into production, consumption, and losses of soil resources, and bridge gaps in soil microbial monitoring that are inherent to -omics methods. Long-term mapping that connects subsurface processes to aboveground flux observations—with explicit resolution of hot-spot and -moment contributions to total soil emissions—will be especially valuable^66^. Measuring soil gases alongside environmental drivers (e.g., oxygen, moisture) in intact soil systems in the field will help uncover in situ soil processes, e.g., dry–wet cycles^49^, that depend on soil structure and are not fully represented in laboratory studies. The system described here is amenable to long-term field deployment with minimal operator intervention (Supplemental Information).

### Versatile applications

Integrating existing trace gas analyzers with soil gas probe sampling systems offers unique insights into myriad subsurface processes and systems. The tools presented in this work open up new windows into subsurface N, C, and other macronutrient transformations that can provide actionable information to optimize agricultural productivity (i.e., precision agriculture) and minimize the contribution of soil emissions to regional and global greenhouse gas budgets. Moreover, new probe technology can be extended to subsurface pollution remediation and control efforts to provide real-time information on system behavior and treatment efficacy. Given the prevalence of gaseous signatures of microbial and abiotic processes across environments and systems, the technical advances demonstrated here for subsurface gas sampling with diffusive probes provide new perspectives into critical and diverse applications.

## Materials and methods

### Probes

We designed novel soil gas sampling probes from porous sintered PTFE (sPTFE). The molecular weight and C-F bonds make PTFE non-reactive (chemically and biologically inert), insoluble, and able to withstand harsh conditions^43^. While ePTFE is expanded through a mold and typically suffers from non-uniform pore distributions and pore sizes < 1 µm, the sintering process retains PTFE’s chemical resistance and results in uniformly distributed pores > 10 times larger than ePTFE, allowing fast diffusion across probe walls. Contrary to ePTFE, the mechanical strength of sPTFE allows self-supporting designs. Soil probes in this study were made from sPTFE blocks (Berghof GmbH, Eningen, Germany) that were machined (White Industries, Petaluma, CA, US) into tubes. Probes were 9.5–12.7 mm outer diameter (OD), 6.4–9.5 mm inner diameter (ID), 150 mm length (L) and had characteristic pore sizes of 8, 10 or 25 µm^42^. The prototype probe assemblies used stainless steel reducing unions to connect the sPTFE tube inlet and outlet to 1/8″ FEP tubing in a flow-through design. No water breakthrough, evidence of biofouling, or structural failures were observed during these experiments.

### Trace gas instruments

Tunable Infrared Laser Direct Absorption Spectroscopy (TILDAS) instruments (Aerodyne Research, Inc., Billerica, MA) were coupled to the soil gas probes. These analyzers use high-resolution infrared spectrometry to quantify trace gases such as N_2_O, CH_4_, CO, CO_2_, NO, NO_2_, and other species^67,68^. For the soil probe application reported here, we developed a dual-laser TILDAS instrument capable of simultaneously measuring ^13^C-CH_4_ and ^12^C-^12^CH_4_ at 1294 cm^-1^, and ^15^N^14^N^16^O, ^14^N^15^N^16^O, ^14^N^14^N^18^O, and ^14^N^14^N^16^O at 2196 cm^-1^. Precisions at ambient concentrations of 1.4‰, 2.2‰, and 0.2‰ in 2 min (Allan-Werle variance minimum) were achieved for both variants of δ^15^N-N_2_O, δ^18^O-N_2_O, and δ^13^C-CH_4_, respectively, where

$${\delta }_{i}X=({R}_{n}-1)\times 1000$$

R_n_ refers to the ratio of the rare isotopologue, ^i^X, to its abundant isotopologue ^69^. The precision of δ^15^N_bulk_ and δ^15^N_SP_ at 325 ppb was 0.9‰ and 1.6‰, respectively. A second dual N-suite TILDAS (Aerodyne Research, Inc.) was placed in series to measure NO at 1900 cm^-1^ and NO_2_ at 1626 cm^-1^, with precisions of 120 and 70 ppt in 1 s, respectively. Calibrations were performed for each trace gas and isotopomer (Supplemental Information). Infrared N_2_O isotopic measurements can be influenced by matrix effects expected in the unique composition of the soil gas matrix: elevated CO_2_ and H_2_O, low O_2_^70^. We quantified potential matrix effects over the observed ranges of CO_2_ and H_2_O concentration and between 0% and 20.9% O_2_. We corrected reported N_2_O isotope values for CO_2_ and H_2_O effects and included the potential effect of variations in O_2_ in measurement uncertainty (Supplemental Information).

The TILDAS isotopic platform was modified to reduce the sample volume demand. The TILDAS absorbance cell initially had a volume of 485 cm^3^, leading to a turnover time of ~ 30 s when operating in a continuous sampling mode with a 50 sccm intake flow and a cell pressure of 20–30 Torr. Long turnover times would translate to undesirably long time intervals between measurements and a larger removal of soil gas by diffusion into the sampling stream. We designed a 3D-printed volume reducing insert for the 76 m cell (Fig. S1) with interior walls that follow the contour of the multipass pattern envelope, maintaining the 76 m pathlength while reducing the cell volume from 485 to 245 cm^3^ and cell time response by 25%. After printing the insert using PA2200 nylon, the interior and exterior surfaces were sealed with urushi lacquer—a stable, durable, inert coating^71^.

### Sampling and sample transfer system

**Figure 5:**
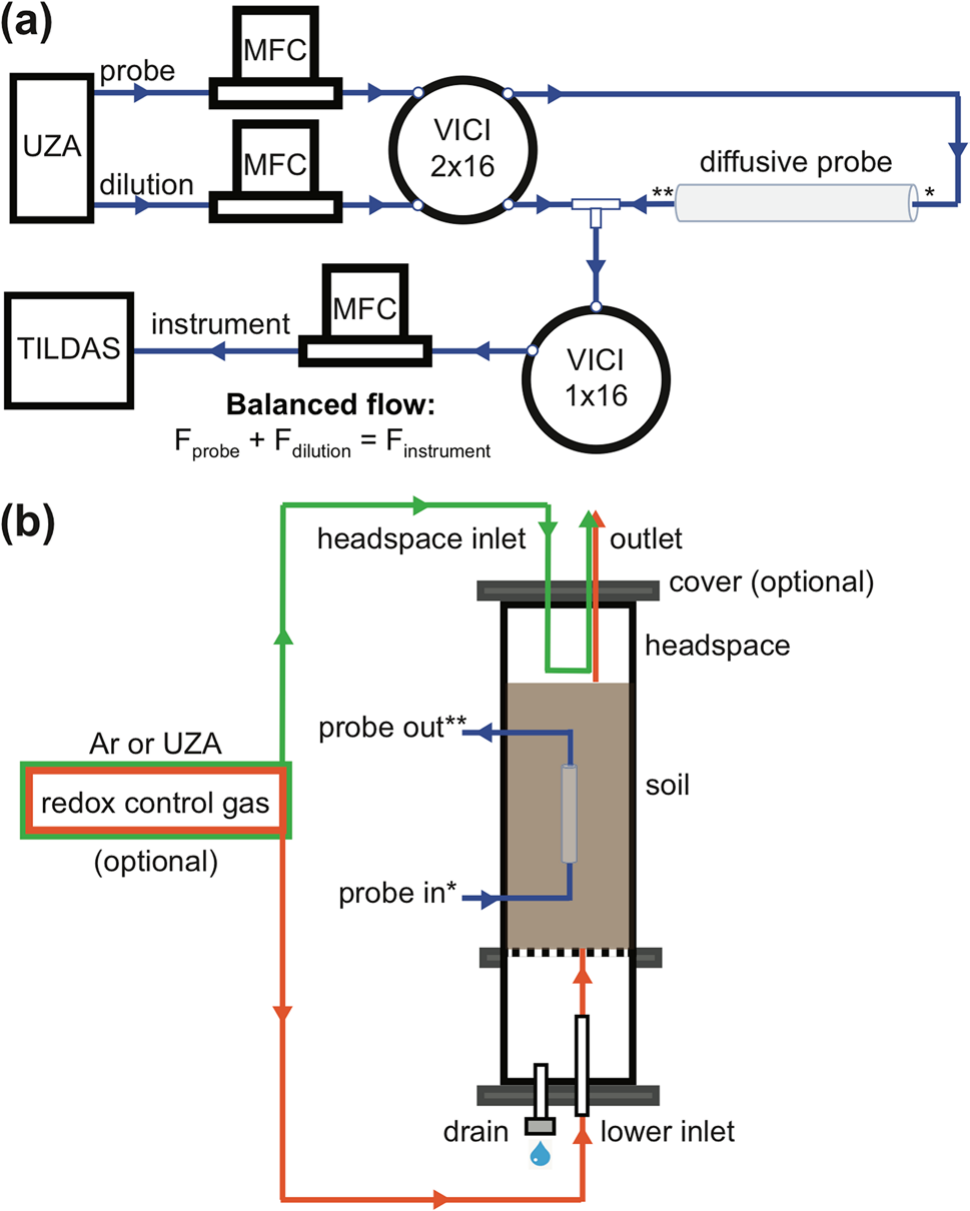
(**a**) Probes sample gases by diffusion across a sPTFE membrane into ultra zero air (UZA) flowing through the probe. Probe outflow is diluted. Flow is balanced—mass flow controllers (MFCs) control flow into the instrument at a rate equal to the sum of probe and dilution flows—to prevent advective flow across the membrane. Two VICI valves select the probe to sample (one of sixteen possible probes illustrated in this setup). (**b**) Lab-based soil columns to demonstrate probe performance (asterisks cross-reference plumbing in (**a**)). Columns allowed wetting from above and drainage of excess water through sealable port at base. Redox state of soils could be controlled by Ar or UZA through-flow through the lower inlet (orange lines) and maintained with optional column cover and a headspace flow (green lines).

The sampling system was operated in a continuous flow arrangement. Equilibrated gas was sampled directly from the soil probe in a flow-through configuration with online dilution at the soil probe outlet (Fig. 5a) to avoid water condensation and increase gas volume delivered to the analyzer(s). We built a custom sample transfer system that allowed integration of multiple probes with a TILDAS (Fig. 5a). Controlled gas flow was directed through the system via 1/8 PFA tubing using multi-port sample selection valves (VICI Valco, Houston, TX, USA). The system used two mass flow controllers (MFC; Alicat Scientific, Inc., Tucson, AZ) to regulate streams of ultra zero air that were delivered in tandem through a 16 × 2 port VICI valve to the selected gas probe inlet and to a ‘T’ connection to dilute the gas probe outlet stream. A 16 × 1 VICI valve was used to collect the total flow, selecting the same probe as the 16 × 2 valve, and the diluted probe sample gas was delivered to the TILDAS at a flow rate controlled by a third MFC at the instrument inlet. Probe inlet and outlet flow rates were matched to prevent advective flow across the diffusive membrane. MFCs were set to maintain accurate flow at probe inlet, dilution, and total flow arriving to the instrument of 16, 29 and 45 sccm, respectively. Ultra zero air was used to balance the probe inlet and outlet flow to transfer samples without advective flow across the probe membrane. However, in some soil environments the diffusive addition of this air to the soil may impact microbial behavior, and future work will explore the use of inert trace gases to mitigate this effect (e.g., He).

Sampling system control was coordinated using the TILDAS instrument software, TDLWintel, which uses an external command language (ECL) to implement valve control, background scheduling, changing TDLWintel operational parameters, and the control of external multiport valves. Commands were combined into scripts that were executed on a schedule for automated and unattended operation for many days at a time.

### Columns

Soil columns were designed for controlled soil manipulation experiments and, in related work, initial evaluation of soil probe performance under controlled soil gas conditions in artificial media^42^. Three 28-L soil columns were constructed from schedule 80 PVC pipe and were 20.2 cm in diameter and 87.6 cm in length (Fig. 5b). At 32.5 cm height a type 304 stainless steel wire cloth mesh (325 × 325 mesh (44 μm), 0.002" opening size) separated the lower section from the soil region to allow soil drainage. Probes were mounted vertically in the center of the columns with sufficient distance from the walls (10 cm) and bottom (15 cm) to avoid edge effects. PTFE and polyetheretherketone (PEEK) bulkhead fittings (IDEX Health & Science LLC., Oak Harbor, WA) provided air- and water-tight connections through column walls (Fig. 5b) and connected the probes to the sampling system. Each column had an optional top cover with ports for flow-through headspace flux measurements, and ports on the bottom plate for input of a controlled advective flow of gas into the soil.

### Experimental design

We performed two soil manipulation experiments in the columns to demonstrate the performance of the soil probe measurement system: (i) Experiment 1—reproducibility of the response of three soil mesocosms to fertilizer addition; and (ii) Experiment 2—comparison of the response to nitrogen addition under aerobic and anaerobic soil conditions. The columns were filled with dry (air-dried) commercial grade topsoil (Timberline Topsoil, Old Castle APG, Atlanta, GA). Subsurface gases were sampled hourly by the probes for over 3 weeks, from filling of columns to the end of the experiments. After allowing the soil to settle for one day following placement in the columns, 4.1 L distilled water was added to each column in ~ 500 mL increments, until water drained out the bottom (Fig. 5b). Four days after soil wetting, Experiment 1 was initiated (Experiment 1, day 0). Commercial fertilizer (24–8-16 N-P-K, Miracle-Gro, Marysville, OH) containing reduced nitrogen (20.5% urea nitrogen and 3.5% ammoniacal) was added to each column at the recommended dosage (4.1 g/L) with an equivalent of 5 cm of irrigation.

Fifteen days after Experiment 1 day 0, the columns were irrigated with distilled water in preparation for Experiment 2. Two days later, the mesocosm in Column 1 was forced into an anaerobic condition by flowing Argon (Ar) gas (500 sccm) through the soil column via the lower column inlet gas port (Fig. 5b). Excess Ar was released via the top of the open column. After 3.5 h, we stopped the Ar flow at the base of the column, the column was capped, and we maintained a low flow of Ar through the headspace of the column to prevent atmospheric oxygen diffusion into the soil. Fertilizer was added to both Columns 1 and 2, as in Experiment 1, to initiate Experiment 2 (Experiment 2, day 0), 17 days after Experiment 1 started. On day 3 of Experiment 2, we stopped the Ar flow to Column 1 headspace and flowed ultra zero air (UZA) through the column via the lower port (500 sccm) for 2.3 h to restore aerobic conditions. Detailed timelines and experimental setup are given in Supplemental Table S1 and Supplemental Figure S4, respectively.

Concentration and instrument diagnostic time series were analyzed in Igor Pro (Version 7, WaveMetrics, Portland, OR) as described in SI. All data and scripts are posted in the Open Science Framework repository at https://osf.io/2ph7s .

## Supplementary Information

Supplementary Information
