# Supplementary material for: Soil gas probes for monitoring trace gas messengers of microbial activity
Source: Sci Rep. 2021 Apr 15;11:8327. doi: 10.1038/s41598-021-86930-8 (PMC8050213; doi:10.1038/s41598-021-86930-8)
Supplement: Supplementary file 1 — Supplementary Information [file 41598_2021_86930_MOESM1_ESM.pdf]

Title: Soil gas probes for monitoring trace gas messengers of microbial activity

Running Title: Soil gas messengers of microbial activity

List of Authors:
Joseph R. Roscioli[1,*], Laura K. Meredith[2,3,*], Joanne H. Shorter[1], Juliana Gil-Loaiza[2], Till H. M. Volkmann[3,4]

Supplemental Information

Supplemental Materials and methods

3D printed insert

We have previously constructed volume reducing stainless-steel inserts for the Aerodyne 36m and 210 m sample cells. Here, we designed and constructed a new cell volume reducing insert for the 76 m cell, which was 3D printed using PA2200 nylon. The interior of the insert was designed to follow the contour of the multipass pattern (Fig. S1). We thereby reduced the cell volume from 485 to 245 cm^3. After printing the insert, the interior and exterior surfaces were sealed with urushi lacquer. Urushi is a stable, durable lacquer that has been used by artists, mostly in East Asia, for thousands of years (McSharry, 2007).

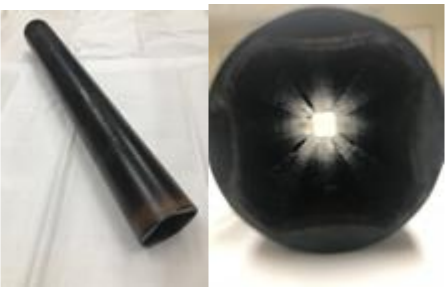

Figure S1. Left: external view of cell insert for 76 m Aerodyne spectrometer. Right: internal view, showing the narrow volume allowed for the laser beam to pass through.

Automation/TDLWintel

Data collection was automated through the use of TDLWintel External Command Language (ECL) scripts. For example, a script that controlled the probe in column #1 included control of all valves, collection of a background spectrum, selection of the correct VICI positions for probe or headspace measurements, and wrote the appropriate “flag” in the data stream to identify what state the sampling system was in. The scripts were run via TDLWintel on an operator determined schedule. A sample script for probe 1 is given below.

```
// script to run background and then measure sample from Probe 1
ca0     //  stop writing index.  This script is for instrument flow of 50 sccm
bz11    // next stc index = 11 for column 1 probe
ba7     // move vici 1 to position 7 to have room air for vocus
ba24    // move vici 2 to position 8 to allow vent of flow during bgs
ano1
```

```
bc1
ano4
aq      // initiate abg
bc345  // wait for bg.  abg is set with 150s flush and 45 sec duration
anc4   // close valve 5
bc1
anc1   // close valve 2
ba17   // move vici 2 to col 1 dilution probe.  outer pos 1 on vici
ba1    // move vici 1 (16x1) to col 1 probe sampling.  port 1
bc2    // wait 2 sec to be sure vicis are all set
ca1    // start marking data with new index
bc600  // PROBE  wait 10 min while sampling probe
```

A soil gas sample was acquired from each probe once per hour, for 10 minutes. The control and measurement script for each probe includes 6 minutes for flushing of the sample cell with UZA and collection of a background spectrum, followed by sampling of the soil gas from the designated probe for 10 minutes. Each script starts with moving the vici valves to an open, non-probe position, opening valves on the sampling lines to allow UZA flow into the instrument, and initiating TILDAS backgrounding. The backgrounds included 150 sec of flush time flowed by 45 sec of measurements and averaging, and then 150 sec flush time (345 sec total). After closing valves that accessed the UZA, the system switched to sampling a probe for 10 minutes, with both dilution flow and flow through the probe initiated. The entire process resulted in ~16 minutes per probe, for 3 probes, and a calibration period (12 minutes), resulting in a minimum return period of 1 hour. Longer averaging for better precision, or more frequent calibrations would lengthen this. Before addition of nitrogen, probe sampling was halted for 12 hours then restarted. The measured N_2O concentration in the columns immediately after restarting were within 1% of the subsequent hourly samples, indicating that the soil was fully recovering within the hour between samples.

TILDAS

The TILDAS platform (Aerodyne Research, Inc., Billerica, MA) operates by drawing an air sample into an absorption cell. Laser light travels through the cell in a multipass configuration, resulting in an effective absorption pathlength of typically 36, 76 or 204 m. The laser wavelength is scanned at kHz rates over the rovibrational absorptions of the molecules of interest, creating transient reductions in light level that are detected on a cooled infrared detector [1]. These light absorptions are fit to known profiles to determine each molecule's concentration in real time using proprietary acquisition and analysis software, TDLWintel (Aerodyne Research, Inc.). The N_2O/CH_4 isotopomer TILDAS was configured with two quantum cascade lasers (QCLs) (Alpes Lasers, St-Blaise, Switzerland) for 1294 and 2196 cm^{-1} for CH_4 and N_2O, respectively, a 9 µm mercury cadmium telluride (MCT) detector from Vigo®, and a 76m multipass absorption cell.

Calibration

We obtained limited noncommercial calibrated N_2O reference gases (Shuhei Ono, Massachusetts Institute of Technology, Cambridge, MA) for intermittent manual isotopic

calibrations. The isotopic ratios of the pure samples of N_2O (MIT Ref I and II) have been determined by IRMS and TILDAS measurements externally verified (S. Toyoda, Tokyo Institute of Technology, Tokyo, Japan) [2]. From the pure samples, we made a 6 L surveillance standard of ~1000 ppm N_2O with the MIT Ref II as the TILDAS N_2O isotopologue calibration standard against which we measure the concentration dependence of the isotopic ratios. These dependencies were generally <1 per mil per ppm of N_2O up to 30 ppm N_2O.

Lower atmospheric isotopic ratios of N_2O tend to be relatively stable [3], with a $\delta^{15}N_{bulk}$ value of 6.3-6.7‰, $\delta^{15}N_{SP}$ of 18.7‰ [4], and $\delta^{18}O$-N_2O value of 44.4‰ [3]. After calibrating against the limited MIT reference, we found that the isotopic ratios observed in ambient air were within 3‰ of the expected atmospheric values. The measured atmospheric N_2O isotopic values were remarkably stable, drifting by 2.2, 0.8, and 0.2 per mil for $\delta^{15}N_{bulk}$, $\delta^{15}N_{SP}$, and $\delta^{18}O$-N_2O, respectively over the course of the experiment (derived from a linear fit of the measured values). Because of this stability, during routine measurements we relied on hourly measurements of ambient air as an in situ isotopic surveillance standard, and used the mean of the calibration factors over the course of the experiment. Uncertainties in isotopic shifts due to calibration drift are calculated to be +/- 1.1‰, +/- 0.4‰, and +/- 0.1‰ for $\delta^{15}N_{bulk}$, $\delta^{15}N_{SP}$, and $\delta^{18}O$-N_2O, respectively.

Concentrations of N_2O, CH_4, and CO_2 were based upon the HITRAN database and agree with ambient values to within 1%, 2%, and 10%, respectively. Concentrations of NO and NO_2 were calibrated using known high concentration standards diluted into ultra zero air.

Matrix Effects Measurement and Correction

Infrared measurements of N_2O isotopes are known to exhibit artifacts due to the presence of other species in the gas matrix[5]. These artifacts are primarily derived from two sources: i) the collisional pressure broadening parameters of the N_2O isotopologues change differently when the gas matrix changes; and ii) spectral absorptions in the infrared scan are unaccounted for, leading to direct spectral interference. These two sources manifest differently in their impact upon the isotopic ratios. The pressure broadening effect depends upon the degree to which the gas matrix changes, but is independent of the N_2O concentration ([N_2O]), whereas spectral interference effect is dependent upon the both the gas matrix change and the N_2O concentration. We observed both of these effects when exploring the impact of enhanced H_2O and CO_2, and depleted O_2 (e.g. anaerobic soil gas) upon the measured isotopic ratios. Generally we found these effects to be <10 per mil, and their impact was further mitigated by 2.5x sample dilution in ultra zero air (UZA).

Matrix dependence tests were performed by sampling a 50 ppm N_2O calibration tank, which served as a stable source of N_2O isotopologues. A small, adjustable flow from this source was entrained into a gas matrix that was formed from combinations of UZA, pure N_2, CO_2, and H_2O. All flows were controlled using mass flow controllers (Alicat, Inc). The TILDAS instrument sampled from the resulting mixture in an overblow configuration. We measured dependences of isotopic ratios with variations of CO_2 in UZA, H_2O in UZA, and N_2 in UZA, for 3-4 N_2O concentrations betweens 400 ppb to 10000 ppb. CO_2 was varied in 3 steps from 0% to 2.5% v/v, O_2 was varied in 3 steps from 0% (dry) to 2.5% (saturated at ~25 C), and the UZA/N_2 ratio was varied to yield O_2 concentrations at 3 steps between 20.9% (ambient) and 0% (fully anoxic). For

every N_2O concentration, triplicate measurements were performed for each step in matrix composition. The TILDAS performed spectral backgrounds using UZA before every round of measurements.

Shifts of isotopic ratios relative to a pure UZA matrix are shown in Figure S2 for varying N_2O concentrations, where by definition no shift (difference of zero) is observed when the gas matrix has not changed (is UZA). All isotopic measurements exhibited a linear relationship with the magnitude of the change in matrix composition (e.g. H_2O, CO_2 and O_2 concentration). The isotopic shifts due to changes in H_2O and CO_2 were dependent upon [N_2O], while the O_2-dependence was largely N_2O independent.

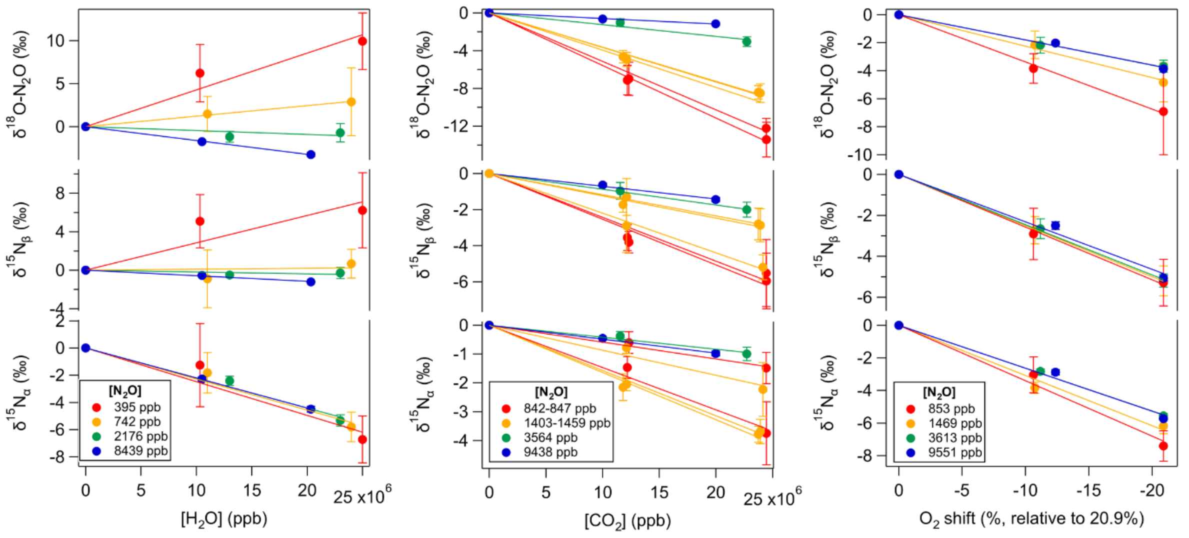

Figure S2. Shifts of $\delta^{15}N_\alpha$, $\delta^{15}N_\beta$, and $\delta^{18}O$-N_2O as a function of changes to the gas matrix by alterations to the presence of H_2O (left), CO_2 (center), and O_2 content (right).

The slopes derived from fits of the data in Figure S2 reveal the matrix-dependence for each isotopic ratio at each N_2O concentration. Linear fits (y=a+bx) of this matrix-dependence vs 1/[N_2O] (Figure S3) reflect the extent to which the matrix effects are derived from spectral interference vs pressure broadening effects. The slope of these fits ("b" in Figure S3) indicate the degree to which spectral interference plays a role, while the fit intercept ("a" in Figure S3) indicates the role of pressure broadening.

The observed slopes in Figure S3 are varied for CO_2 and H_2O (left and center panel), indicating spectral interference is important in some cases. The fits also show significant intercepts (up to 2.2 ‰/% H_2O and 0.6 ‰/% CO_2), indicating that pressure broadening effects are also important. On the other hand, slopes associated with changes in O_2 content (Figure S3, right panel) were small, but the observed intercepts indicate a significant pressure-broadening component (up to 0.2‰/% O_2).

The linear fits derived in Figure S3 were combined with measured H_2O and CO_2 concentrations to correct the isotopic ratios. Oxygen content was not measured during the experiments, but dilution of the sample by 2.5x resulted in a minimum O_2 concentration of 12.5%, and uncertainties of 3‰, 2‰, and 2.5‰ for $\delta^{15}N_\alpha$, $\delta^{15}N_\beta$, and $\delta^{18}O$-N_2O, respectively. Uncertainty in

$\delta^{15}N_{bulk}$ and $\delta^{15}N_{SP}$ were calculated to be 5‰ and 1‰, respectively. Overall uncertainty of the isotopic measurements were calculated by adding the matrix and calibration uncertainties in quadrature.

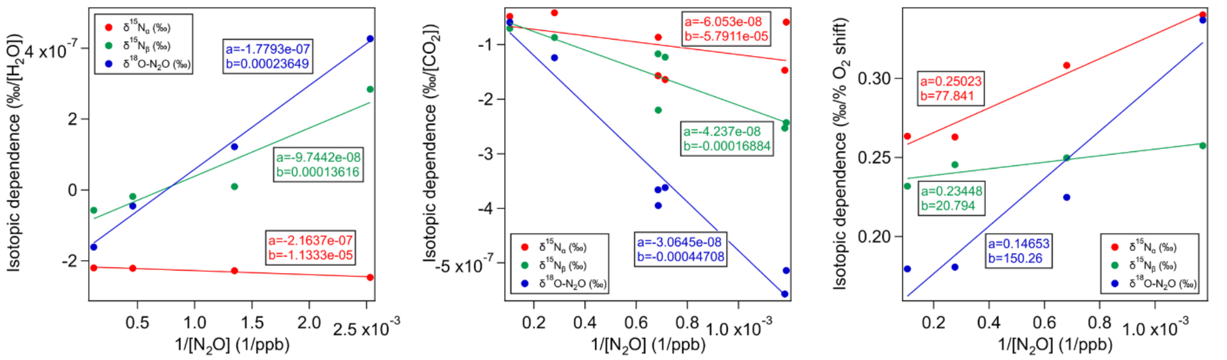

Figure S3. Matrix-dependence of isotopic shifts as a function of $1/[N_2O]$. Linear fits to this data

Field Deployment

While this paper describes laboratory-based studies, the measurement system is amenable to field deployment and has been deployed unattended for months at a time [6,7]. The TILDAS can be housed in a temperature-controlled room or in an Aerodyne weather-proofed, temperature-controlled enclosure. It is important to perform frequent backgrounds to account for any measurement drift due to any remaining temperature fluctuations. The valve system and pump do not need temperature control, but do need to be housed in a ventilated weather-proof enclosure to protect against precipitation.

Other requirements for lab or field measurements include electrical power, and availability of ultra zero air (UZA). UZA consumption varies depending upon the specific flow design being used, but can be as small as 20 L/day.

Table S1. Timeline of treatments in days relative to the N addition of each experiment.*

Experiment	Column(s)	Soil fill (day)	Water only (day)	Redox state	Headspace	End
1	1-3, replicates	-5	-4	Ambient (aerobic)	open	15
2	1	-20	-2	Ar on days 0-3 / UZA on day 3 / no flush on days 3- 6	Ar on day 0-3 / day 3-6 open	6

2	2	-20	-2	Ambient (aerobic)	open	6

*Day # relative to start of Experiment 1 or Experiment 2, where each Experiment start = day of N addition

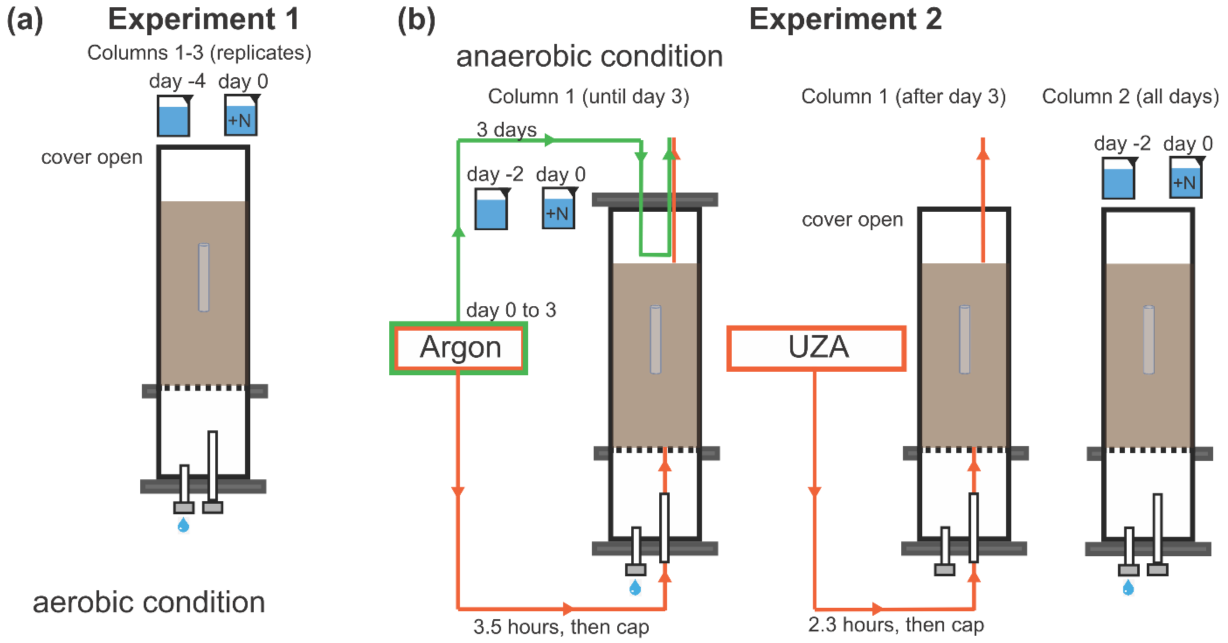

Figure S4. Illustrated experimental treatments (described in Table S1). **(a)** Experiment 1 treated three soil column replicates identically with addition of distilled water (day -4) and fertilizer (day 0) with the column cover open and no use of control gas. **(b)** Experiment 2 treated both columns with distilled water (day -2) and then fertilizer (day 0), but the treatments then diverged for the two soil columns. Column 1 underwent a 3-day anaerobic treatment (whole-column Ar flush from below followed by maintenance of anaerobic conditions by headspace flushing) before rapidly shifting to aerobic conditions by flushing with UZA at the end of the 3 days and then leaving the cover open. In contrast, Column 2 was passively maintained under aerobic conditions with the cover open and no control gas as in Experiment 1.

Correlation of SP and δ448

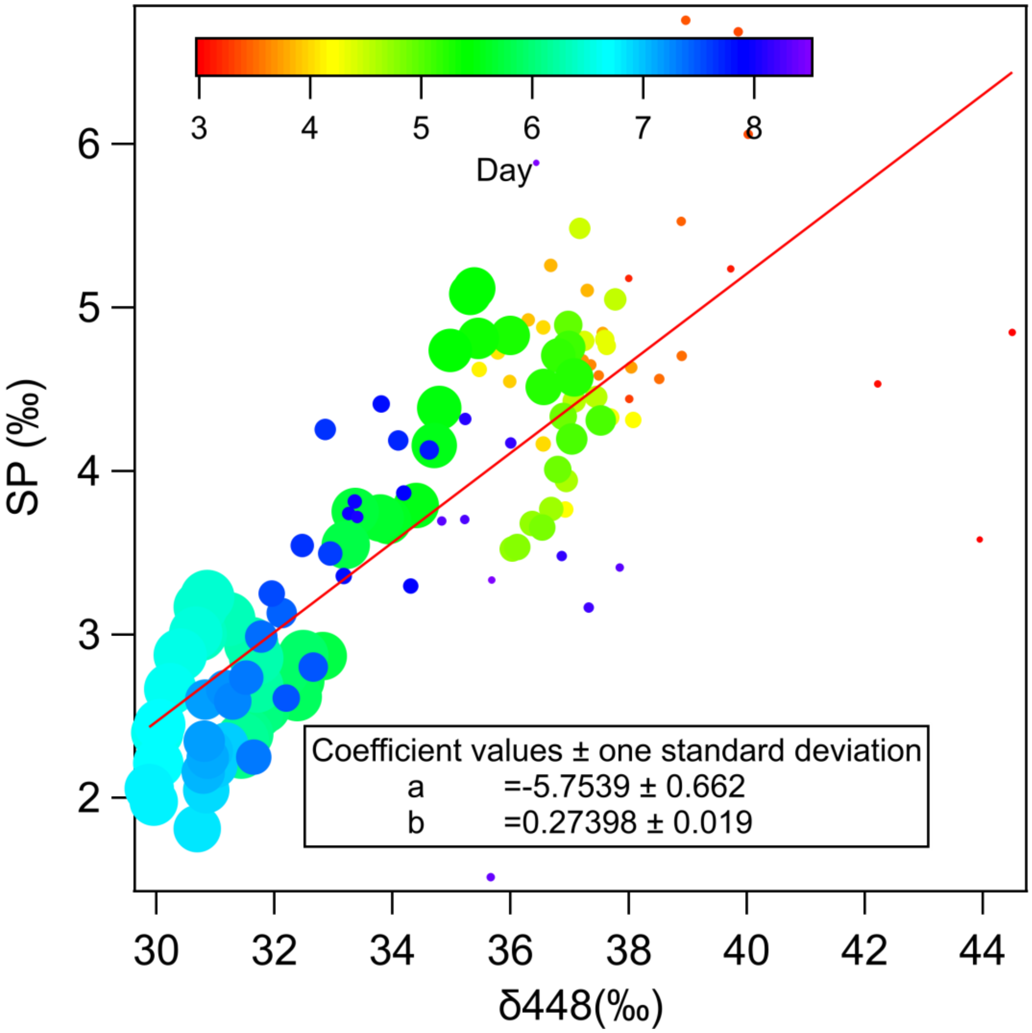

Figure S5. Correlation of SP and $\delta^{18}O$-N_2O (δ448) from day 3.0-8.5 of Experiment 1. Data is average among all 3 columns. Point color refers to the time of measurement (days), and point size is weighted by N_2O concentration. Fit is unweighted.

Correlation between NO and N_2O

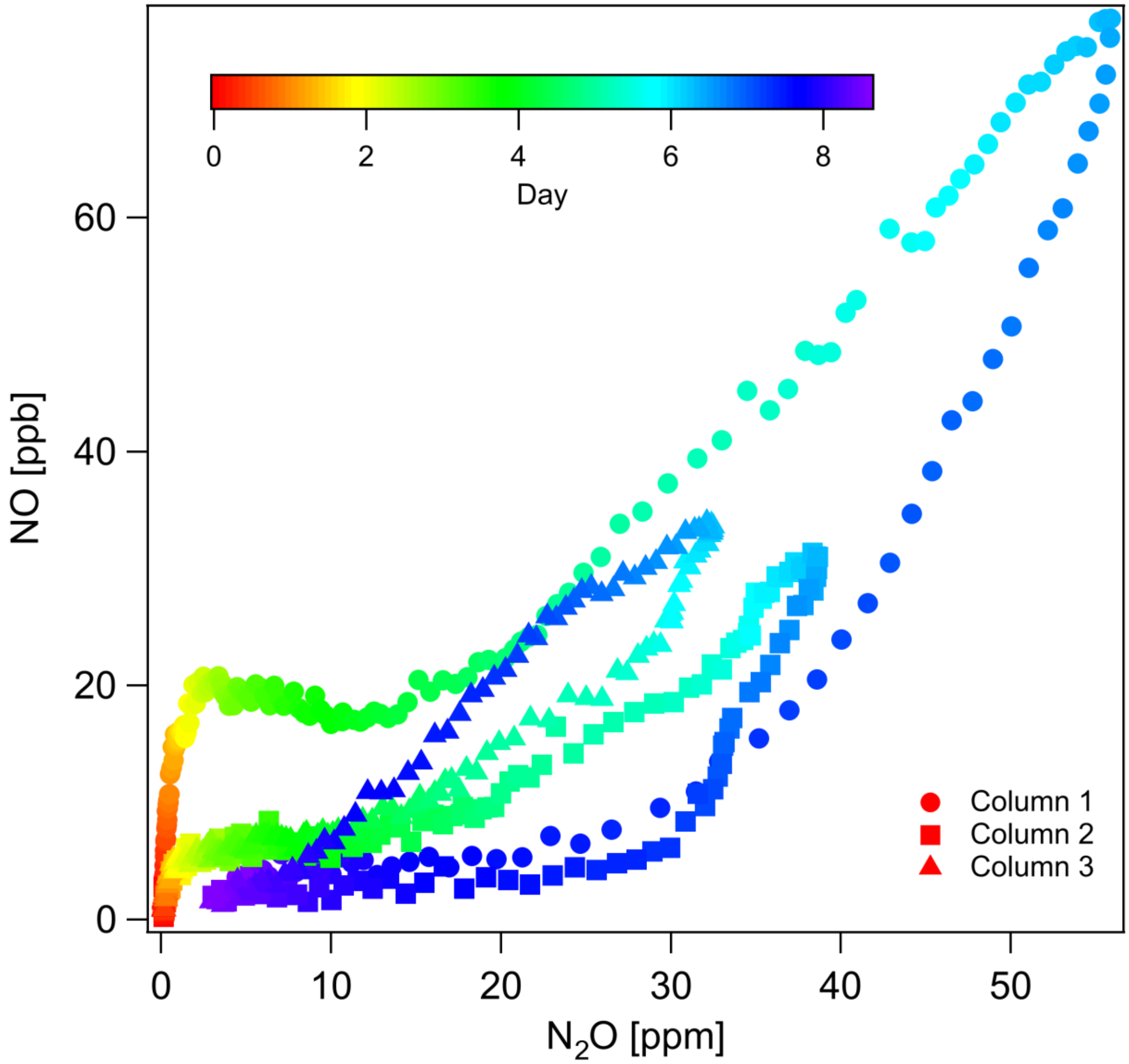

Figure S6. Correlation plot of NO and N_2O over the course of Experiment 1 for Column 1 (circle), 2 (square) and 3 (triangle). Marker color refers to the time of measurement (days). Early NO production (red-orange) is uncorrelated with N_2O, while later NO (green-blue) is correlated. After day 8 (blue-purple), NO and N_2O quickly decrease, resulting in a correlated return to low concentration.

References

1. McManus JB, Zahniser MS, Nelson DD, Shorter JH, Herndon SC, Jervis D, et al. Recent progress in laser-based trace gas instruments: performance and noise analysis. Applied Physics B. 2015. pp. 203–218. doi:10.1007/s00340-015-6033-0

2. McClellan MJ. Estimating regional nitrous oxide emissions using isotopic ratio observations and a Bayesian inverse framework. Massachusetts Institute of Technology. 2018. Available: https://dspace.mit.edu/handle/1721.1/119986?show=full

3. Snider DM, Venkiteswaran JJ, Schiff SL, Spoelstra J. From the ground up: global nitrous oxide sources are constrained by stable isotope values. PLoS One. 2015;10: e0118954.

4. Mohn J, Tuzson B, Manninen A, Yoshida N, Toyoda S, Brand WA, et al. Site selective real-time measurements of atmospheric N2O isotopomers by laser spectroscopy. Atmospheric Measurement Techniques. 2012. pp. 1601–1609. doi:10.5194/amt-5-1601-2012

5. Harris SJ, Liisberg J, Xia L, Wei J, Zeyer K, Yu L, et al. N_2O isotopocule measurements using laser spectroscopy: analyzer characterization and intercomparison. Atmos Meas Tech. 2020;13: 2797–2831.

6. Roscioli. in prep.

7. Meredith. in prep.
